# Supplementary material for: Suppression of magnetic ordering in XXZ-type antiferromagnetic monolayer NiPS3
Source: Nat Commun. 2019 Jan 21;10:345. doi: 10.1038/s41467-018-08284-6 (PMC6341093; doi:10.1038/s41467-018-08284-6)
Supplement: Supplementary file 1 — Supplementary Information [file 41467_2018_8284_MOESM1_ESM.pdf]

Supplementary Information

Suppression of magnetic ordering in XXZ-type antiferromagnetic monolayer $NiPS_3$

Kangwon Kim, et al.

Contents:

- **Supplementary Figure 1.** Determination of the sample thickness.
- **Supplementary Figure 2.** Magnetic susceptibility of single crystal $NiPS_3$.
- **Supplementary Note 1.** Calculation of two-magnon density of states (DOS).
- **Supplementary Figure 3.** Comparison of two-magnon signals of Raman with theoretical calculation of two-magnon DOS.
- **Supplementary Table 1.** Experimental peak positions and calculated phonon frequencies of Raman-active modes of bulk $NiPS_3$ with zigzag antiferromagnetic ordering.
- **Supplementary Figure 4.** Comparison of calculated phonon frequencies with experimental spectra.
- **Supplementary Figure 5.** Temperature dependence of P_5 and P_7 in bulk $NiPS_3$
- **Supplementary Figure 6.** Temperature dependence of Raman spectra of 10 nm, 8L, 4L, 3L, and 2L $NiPS_3$ in cross-polarization scattering configuration.
- **Supplementary Figure 7.** Polarization dependence of Raman spectra of bulk $NiPS_3$.
- **Supplementary Figure 8.** Thickness dependence of polarized Raman spectra.
- **Supplementary Note 2.** Origin of P_3 at ~210 cm^{-1} in few-layer $NiPS_3$.
- **Supplementary Figure 9.** Excitation energy dependence of Raman spectra of 1-3L and bulk $NiPS_3$.
- **Supplementary Figure 10.** Temperature dependence of P_3.
- **Supplementary Figure 11.** Polarized Raman spectra of 10 nm, 8L, 4L, 3L, 2L, and 1L $NiPS_3$

in parallel- and cross-polarization configurations as a function of temperature.

- **Supplementary Note 3.** Extracting transition temperature by using ΔP_2.
- **Supplementary Figure 12.** Extracting transition temperature by using ΔP_2.
- **Supplementary Figure 13.** Comparison of substrate effects.
- **Supplementary Figure 14.** ΔP_2 in $NiPS_3$ on hexagonal boron nitride (hBN).
- **Supplementary Figure 15.** Temperature dependence of quasi-elastic scattering signals.
- **Supplementary Note 4.** Monte Carlo simulations.
- **Supplementary Figure 16.** Results of Monte Carlo simulations.
- **Supplementary Figure 17.** X-ray diffraction patterns of $NiPS_3$ single crystal.
- **Supplementary Note 5.** Degradation test for few-layer $NiPS_3$
- **Supplementary Figure 18.** Photo-degradation test for few-layer $NiPS_3$.

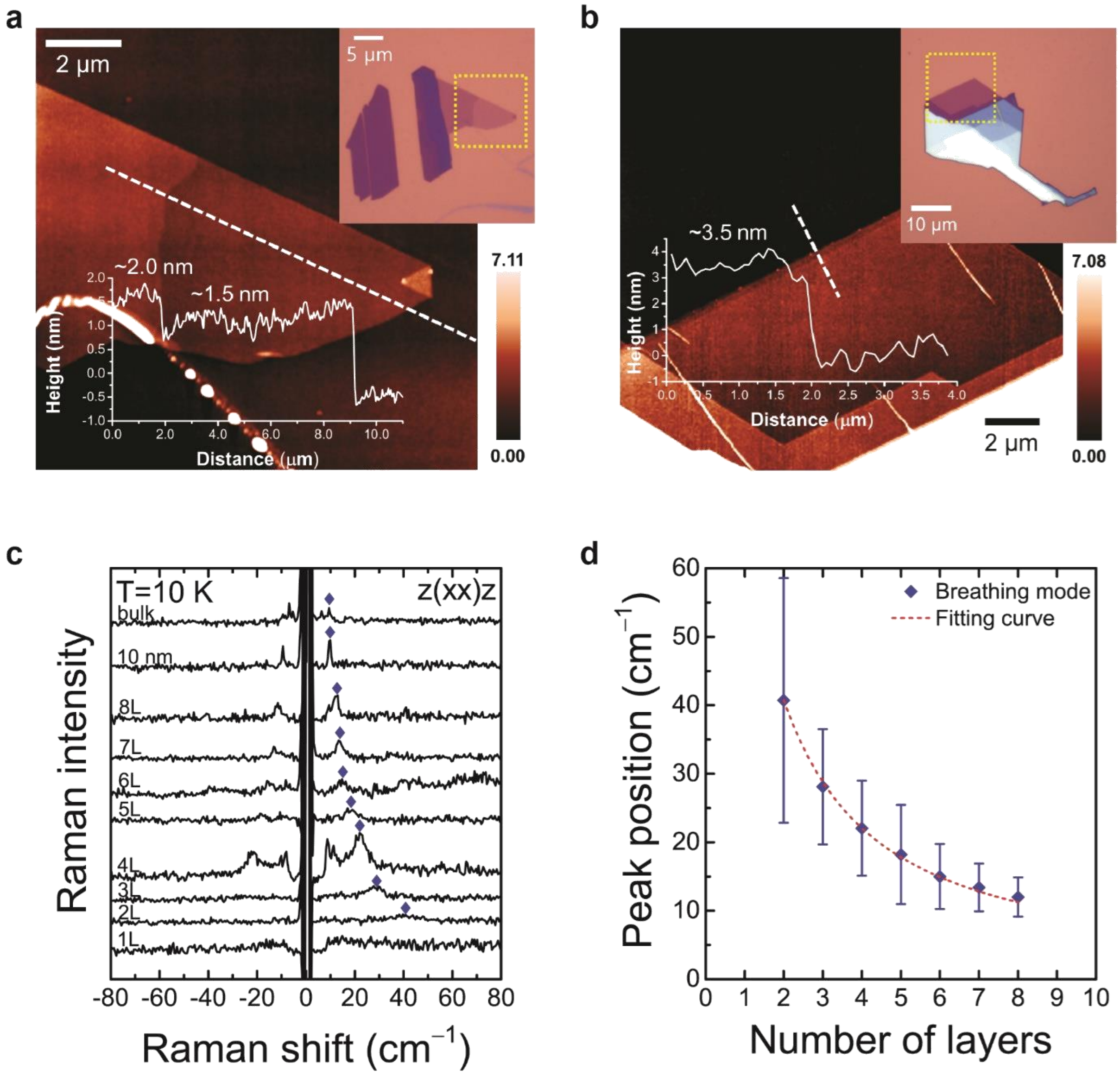

Supplementary Figure 1 | Determination of the sample thickness. a,b, Atomic force microscopy images of 2L, 3L (**a**), and 4L (**b**) $NiPS_3$. The insets are optical microscope images. **c,** Thickness dependence of low-frequency Raman spectra of few-layer $NiPS_3$ at T=10 K. **d,** Position of the breathing mode as a function of the number of layers. The error bars indicate uncertainties due to the peak widths in the spectra. The broken curve is fitting to the linear chain model[1,2].

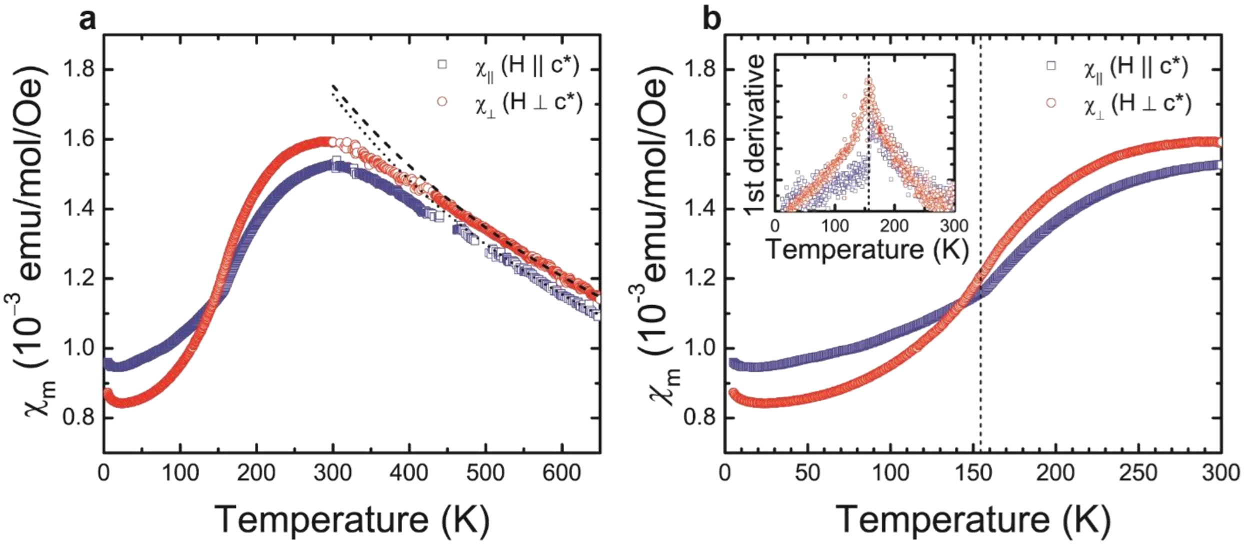

Supplementary Figure 2 | Magnetic susceptibility of single crystal $NiPS_3$. The dashed and dotted curves represent the Curie-Weiss fitting results in **a**. The inset of **b** is the first derivative of the magnetic susceptibility. The peak corresponds to the Néel temperature.

- **Supplementary Note 1**

Calculation of two-magnon density of states (DOS)

We carried out spin waves calculations to explain the two-magnon continuum found in our Raman data of $NiPS_3$. In order to have the zigzag-type magnetic structure as obtained from neutron diffraction studies[3,4], we used a J_1-J_2-J_3 Heisenberg Hamiltonian for the honeycomb lattice. According to previous theoretical studies[5–8] of the J_1-J_2-J_3 model, the zigzag magnetic structure can be stabilized by considering up to third-nearest neighbor interaction (J_3). For example, when there is an antiferromagnetic J_1, a zigzag magnetic structure has been found if both J_2 and J_3 are larger than 0.6 J_1. To be consistent with the experimental evidence suggesting the XY symmetry in the physical properties of $NiPS_3$, we used the following J_1-J_2-J_3 XXZ Hamiltonian as given below:

$$\begin{aligned} H = J_1 \sum_{\langle i,j \rangle} \left[S_i^x S_j^x + S_i^y S_j^y + \alpha S_i^z S_j^z \right] + J_2 \sum_{\langle\langle i,k \rangle\rangle} \left[S_i^x S_k^x + S_i^y S_k^y + \alpha S_i^z S_k^z \right] \\ + J_3 \sum_{\langle\langle\langle i,l \rangle\rangle\rangle} \left[S_i^x S_l^x + S_i^y S_l^y + \alpha S_i^z S_l^z \right] + \sum_i \left[D_1 \left(S_i^x \right)^2 + D_2 \left(S_i^z \right)^2 \right] \end{aligned} \quad (1)$$

First three terms denote the XXZ anisotropic Hamiltonian up to third nearest neighbors with an anisotropy parameter α. The local (x, y, z) coordinates are defined consistently with the magnetic structure of $NiPS_3$ in Supplementary Fig. 3c. The last terms in the bracket are single-ion anisotropies (SIA) along the x- and z-axes, respectively. We neglected an inter-layer coupling as it is known to be smaller by two orders of magnitude than the intra-layer coupling[9,10].

Two-magnon spectrum measured by Raman scattering corresponds to the sum of the two single- magnon with a total momentum of $\mathbf{q}$ = 0. In the magnetic system with negligible magnon-magnon interaction, two-magnon density of states (DOS) can be directly related to the Raman intensity[11]. We calculated the two-magnon DOS in the following way. First, for a given $\mathbf{k}$ point of the two magnon continuum a one-magnon dispersion was calculated at randomly chosen one million sample $\mathbf{q}$ points using the SpinW software[12]. After that, two-magnon DOS was calculated with the following sum rule satisfying kinematic constraints:

$$D_{\mathbf{k}} \left(\varepsilon_{\mu\mathbf{k}} \right) = \pi \sum_{\mathbf{q},mn} \delta \left(\varepsilon_{\mu\mathbf{k}} - \varepsilon_{m\mathbf{q}} - \varepsilon_{n\mathbf{k}-\mathbf{q}} \right), \quad (2)$$

where $\varepsilon_{\mu\mathbf{k}}$ is the dispersion of the μ-th magnon band.

We employed a particle swarm optimization algorithm to find the global minimum in the parameter space to explain the two-magnon continuum observed by our Raman data. The best fitting results were achieved with the following set of parameters: J_1 = 3.18 meV, J_2 = 4.82 meV, J_3 = 9.08 meV, α = 0.66, D_1 = -0.89 meV, D_2 = 2.85 meV (see Supplementary Figs. 3b and 3d).

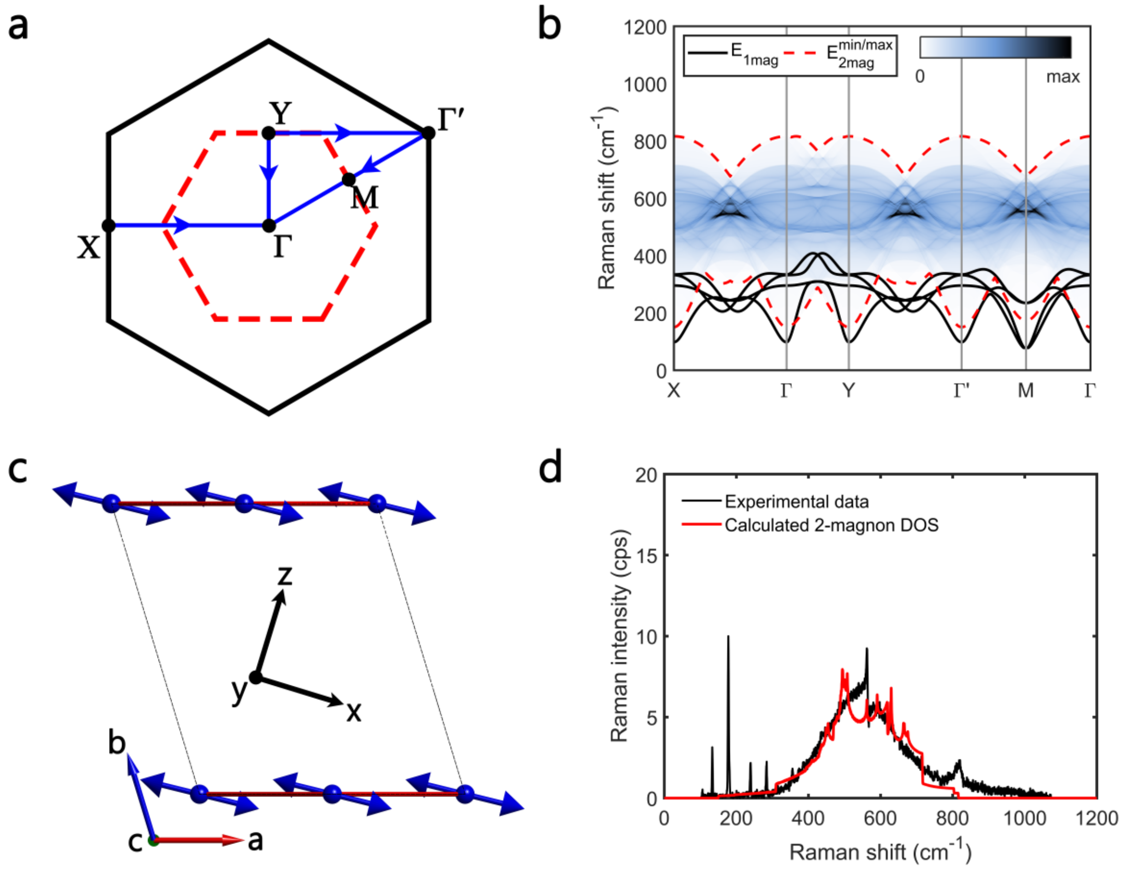

Supplementary Figure 3 | Comparison of two-magnon signals of Raman with theoretical calculations of two-magnon DOS. a, Schematic diagram for the 1st Brillouin zone with several symmetry points marked. **b,** Spin wave dispersion. **c,** Magnetic structure of antiferromagnetic $NiPS_3$. **d,** Comparison of experimental and theoretical two-magnon continuum.

Supplementary Table 1 | Experimental peak positions and calculated phonon frequencies of Raman-active modes of bulk $NiPS_3$ with zigzag antiferromagnetic ordering.

Peak	Experimental (cm^{-1})	Calculation (cm^{-1})	Mode (C_{2h})	Mode (D_{3d})*
		111.04	B_g	
P_1	133.8<br>134.9	117.28<br>118.71	A_g<br>B_g	E_g
P_2	179.0<br>181.3	174.35<br>179.42	A_g<br>B_g	E_g
		213.65	B_g	
P_3	206.7			
P_4	239.6<br>240.1	221.82<br>224.12	A_g<br>B_g	E_g
P_5	256.9	235.56	A_g	A_g
P_6	282.3<br>284.4	269.88<br>270.48	B_g<br>A_g	E_g
P_7	386.1	372.62	A_g	A_g
P_8	446.8			
P_9	564.2<br>565.6	543.47<br>545.07	A_g<br>B_g	E_g
P_{10}	590.5	559.25	A_g	A_g

* Approximate corresponding mode in the D_{3d} point group of monolayer $NiPS_3$.

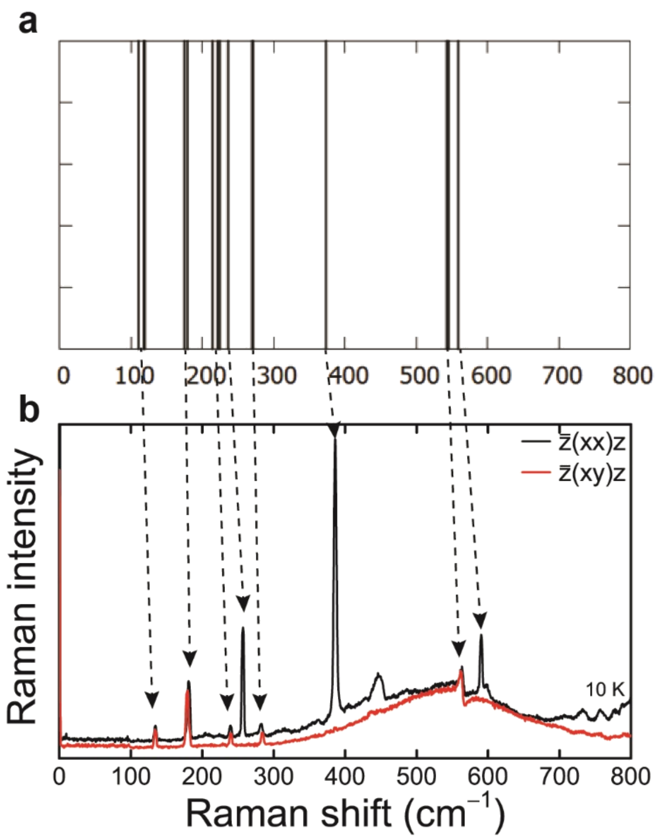

Supplementary Figure 4 | Comparison of calculated phonon frequencies with experimental spectra. a,b, Calculated phonon frequencies of Raman-active modes (**a**) and experimental Raman spectra (**b**) of bulk $NiPS_3$ with zigzag antiferromagnetic ordering.

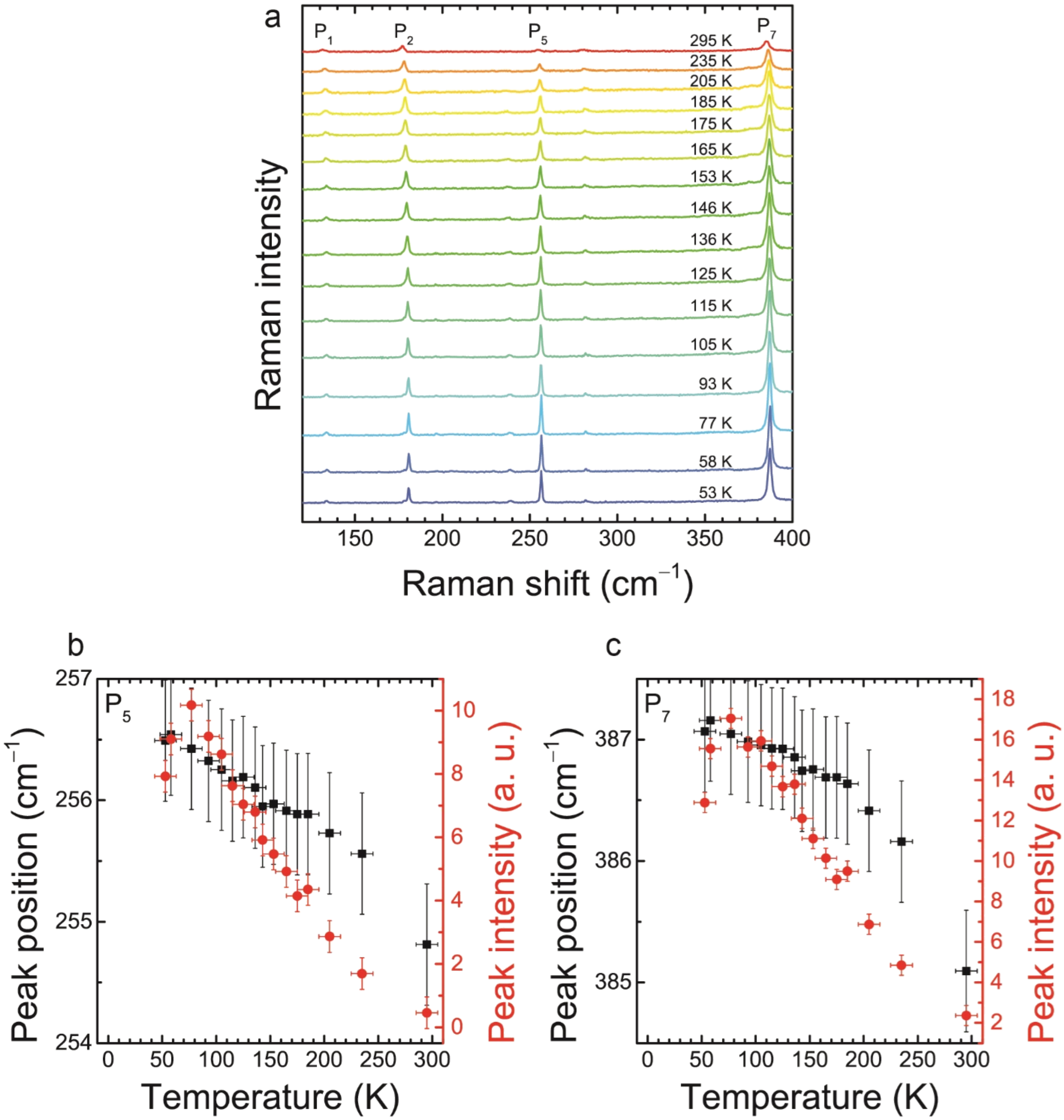

Supplementary Figure 5 | Temperature dependence of P_5 and P_7 in bulk $NiPS_3$. a, Temperature dependent Raman spectra of bulk $NiPS_3$ in $\bar{z}(xx)z$ polarization configuration. **b,c,** Temperature dependence of peak position and intensity of P_5 (**b**) and P_7 (**c**). The error bars indicate experimental uncertainties.

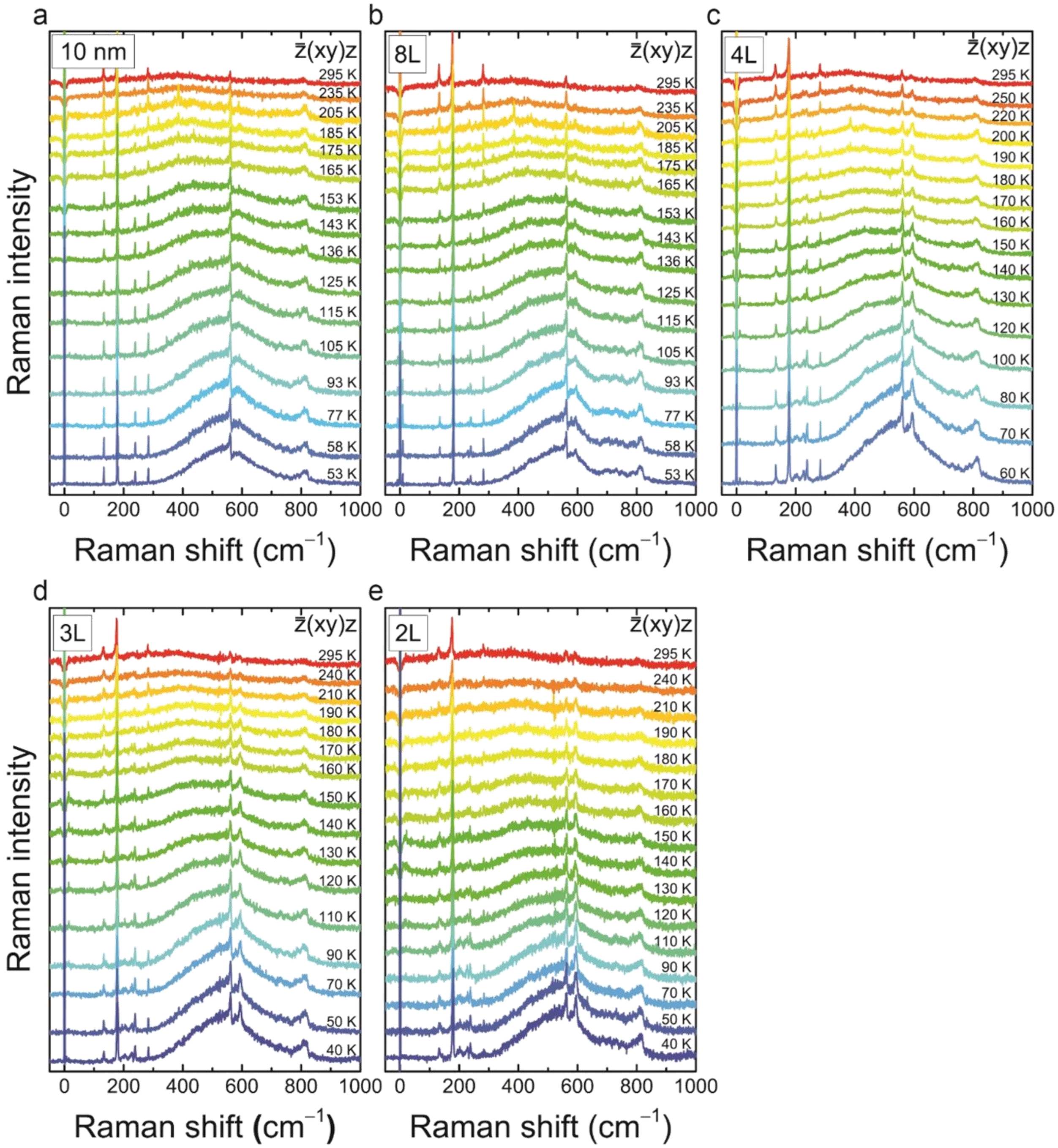

Supplementary Figure 6 | Temperature dependence of two-magnon signal and Fano resonance of P_9. a–e, Raman spectra of 10 nm (**a**), 8L (**b**), 4L (**c**), 3L (**d**), and 2L (**e**) $NiPS_3$ as a function of temperature obtained by using cross-polarization configuration.

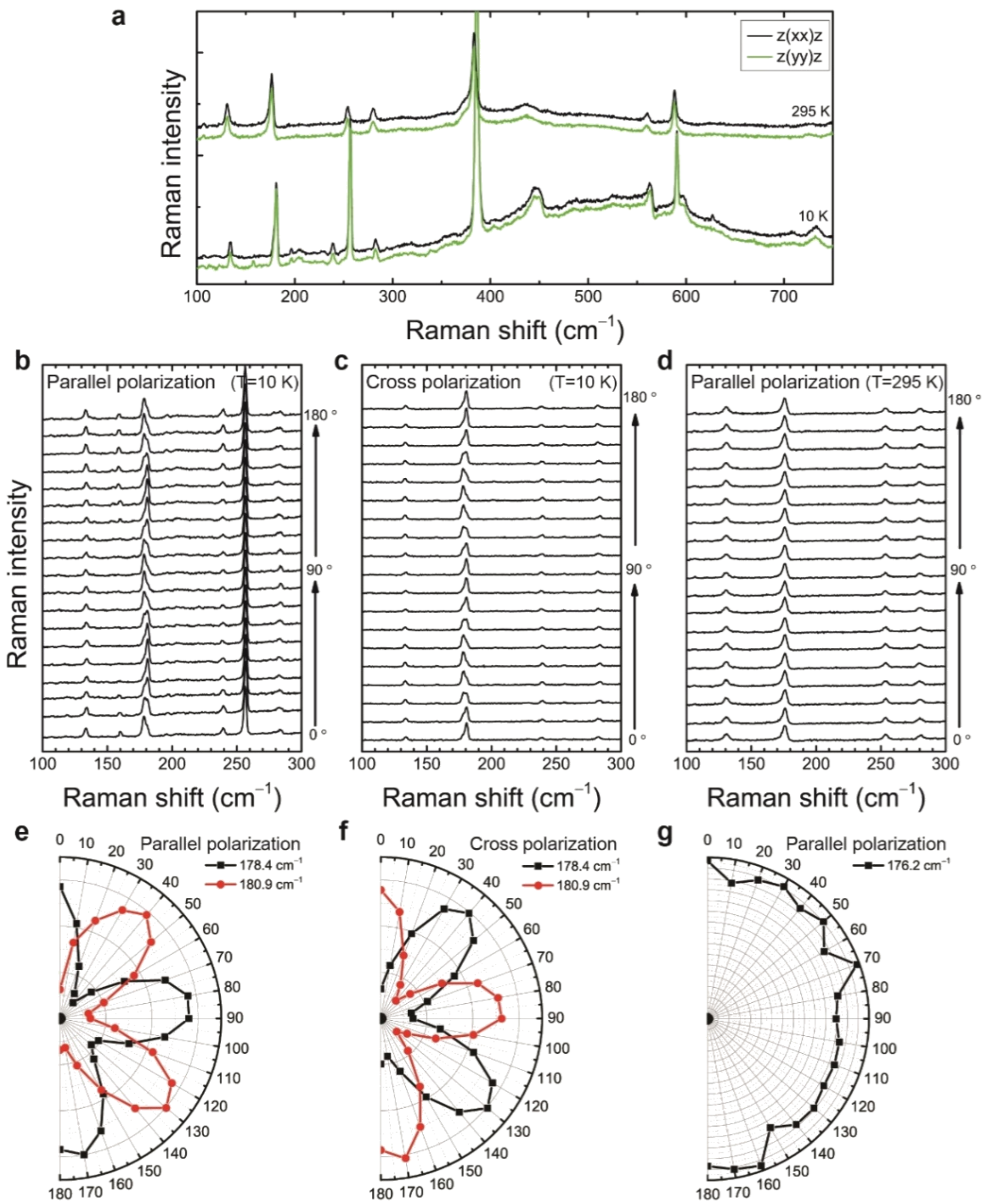

Supplementary Figure 7 | Polarization dependence of P$_2$. a, Comparison of Raman spectra of bulk $NiPS_3$ obtained by using $\bar{z}(xx)z$ and $\bar{z}(yy)z$ polarization configurations shows that *x* and *y* directions are equivalent. **b–d**, Polarized Raman spectra as a function of the incident polarization direction for parallel (**b**) and cross (**c**) polarization configurations at *T*=10 K and for parallel (**d**) polarization configuration at *T*=295 K. **e–g,** Intensities of P$_2$ as a function of polarization at *T*=10 K in parallel (**e**) and cross (**f**) polarization configurations and at 295 K (**g**) in parallel-polarization configuration.

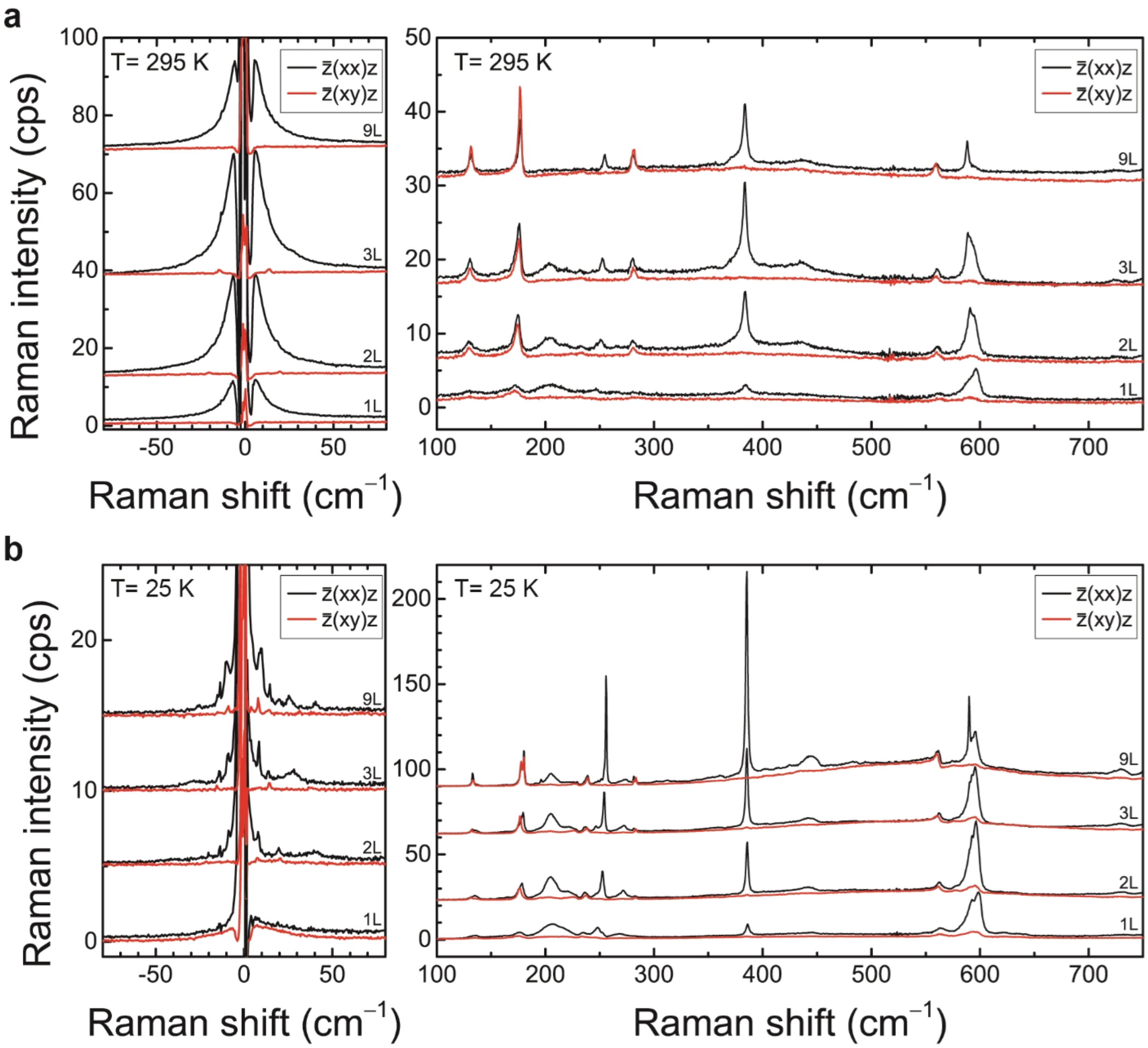

Supplementary Figure 8 | Thickness dependence of polarized Raman spectra. a,b, Polarized Raman spectra of 1L, 2L, 3L, and 9L at T=295 K (**a**), and T=25 K (**b**) in parallel and cross polarization configurations.

- **Supplementary Note 2.**

Origin of P_3 at ~210 cm^{-1} in few-layer $NiPS_3$

A broad and strong peak (P_3) near 210 cm^{-1} is absent in bulk but appears in the spectrum of few-layer $NiPS_3$ (see Supplementary Fig. 8). We interpret that this peak is due to resonance-enhanced multiphonon scattering that is frequently observed in many 2-dimensional materials such as MoS_2 and WS_2[13,14]. For example, in MoS_2, the signal from 2-phonon scattering of zone-boundary longitudinal acoustic phonons (2LA) is strongly enhanced for resonant excitation of 1.96 eV and dominates the spectrum, with an intensity much larger than the main Raman-active zone-center optical phonon modes. This phenomenon has been explained in terms of the interplay between the large densities of states of the zone-boundary phonons and the electronic bands that are resonant with the excitation laser[17].

In the case of $NiPS_3$, theoretical calculations by M. Bernasconi *et al.*[15] predicts that the phonon density of modes is very large near 105 cm^{-1} due to multiple phonon branches near the K point of the Brillouin zone. Resonant enhancement of two-phonon scattering involving these phonons would explain the observed peak at 210 cm^{-1}. In order to verify this interpretation, we carried out Raman measurements on 1-3L and bulk $NiPS_3$ samples using several lasers. As seen in Supplementary Fig. 9, P_3 is present in 1-3L but absent in bulk $NiPS_3$ when the 2.41-eV excitation is used. When the excitation energy is slightly increased to 2.54 eV, P_3 disappears for 2L and 3L and is significantly decreased for 1L. The intensities of the other peaks also decrease, indicating that we are moving away from the resonance, but P_3 is preferentially suppressed, supporting our hypothesis that this peak is preferentially enhanced due to a special resonance conditions. For the 2.81-eV excitation, P_3 is completely suppressed for all samples, but other peaks are also greatly diminished. These observations support our interpretation that this peak originates from two-phonon scattering strongly enhanced by resonance effects.

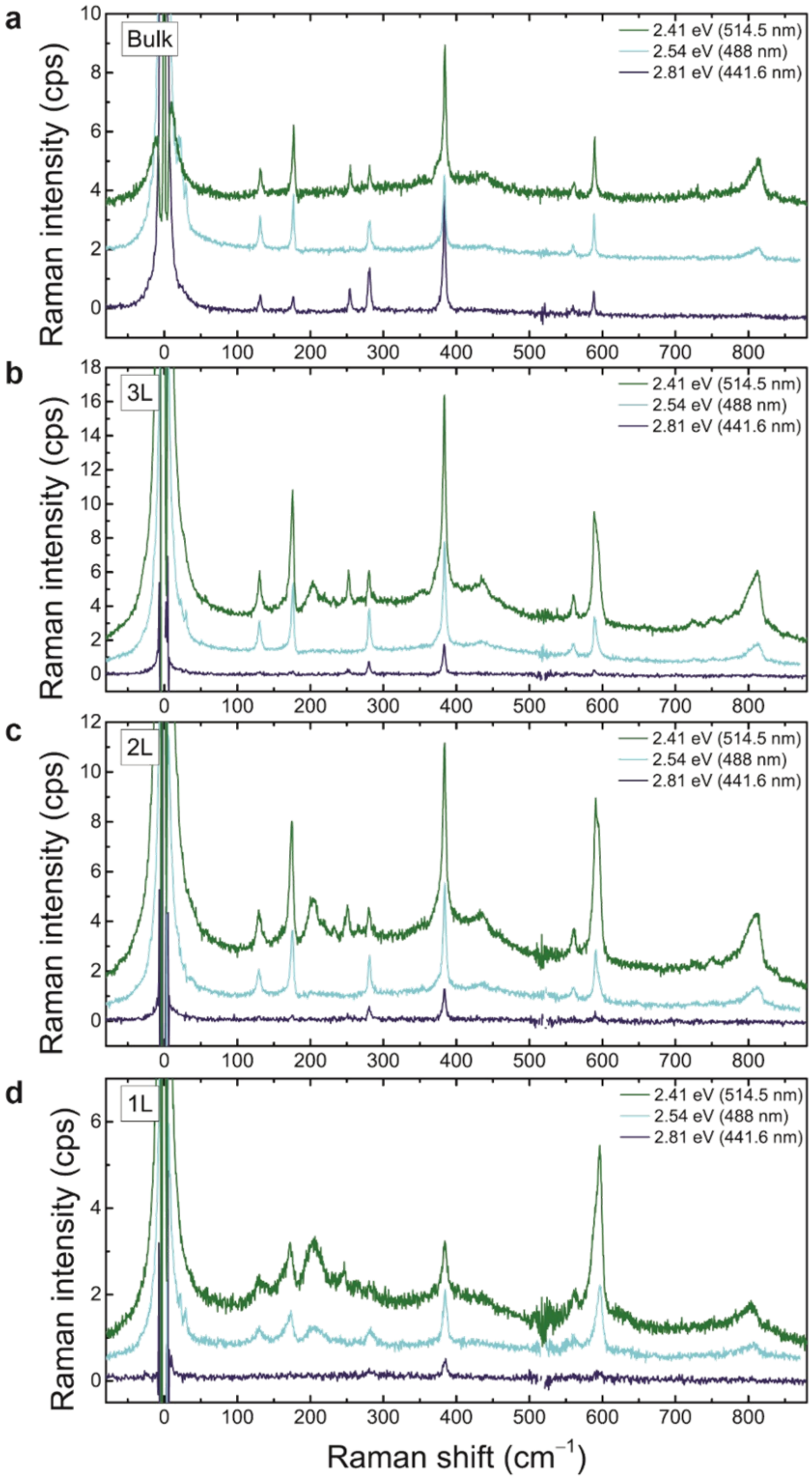

Supplementary Figure 9 | Excitation energy dependence of Raman spectra of 1-3L and bulk $NiPS_3$. Unpolarized Raman spectra measured at room temperature by using the 2.41, 2.54, and 2.81 eV excitation energies for bulk (**a**), 3L (**b**), 2L (**c**), 1L (**d**) $NiPS_3$.

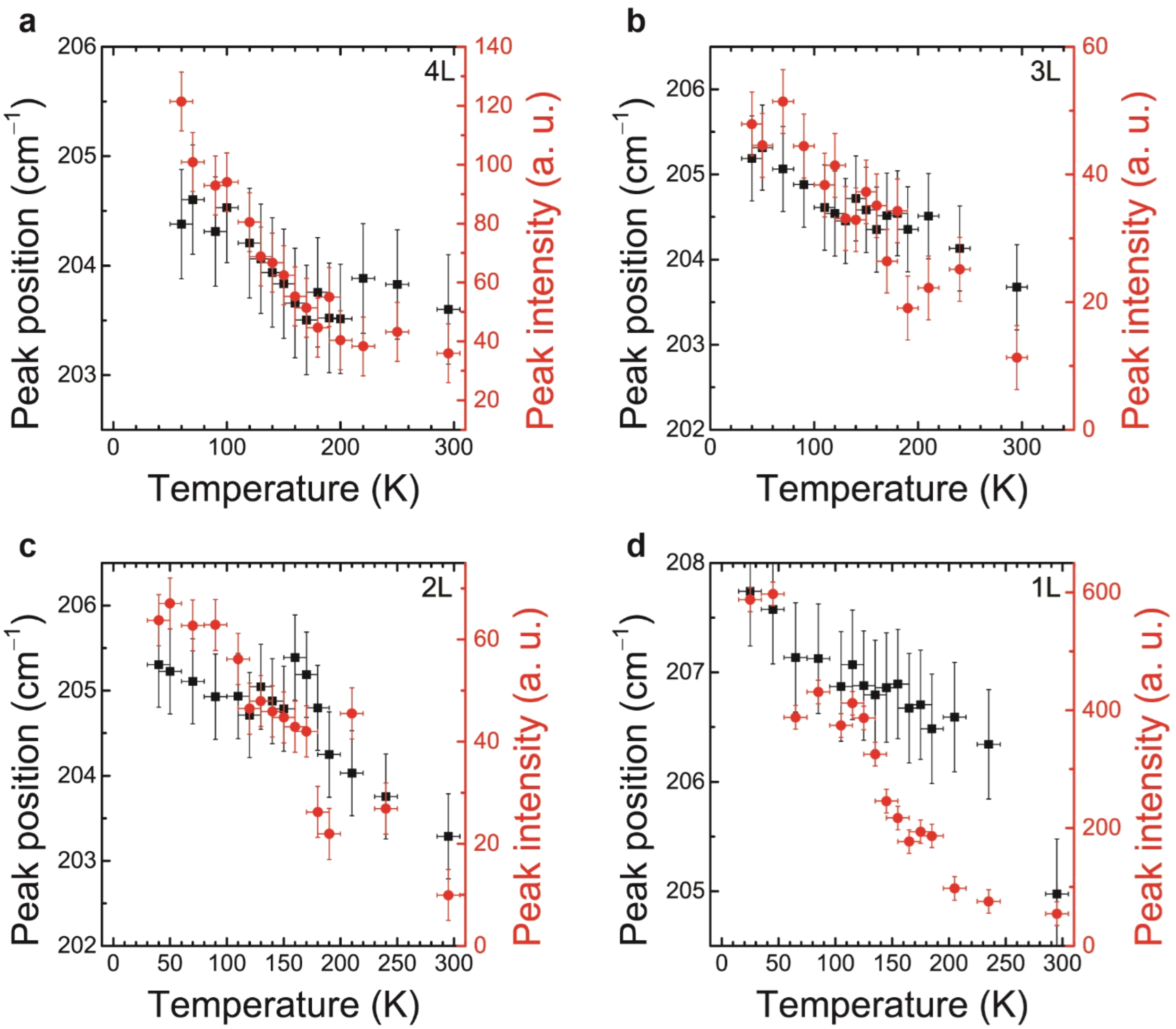

Supplementary Figure 10 | Temperature dependence of P_3. a-d, Temperature dependence of peak position and intensity for 4L (**a**), 3L (**b**), 2L (**c**), and 1L (**d**). The error bars indicate experimental uncertainties. No correlation with the magnetic transition is seen.

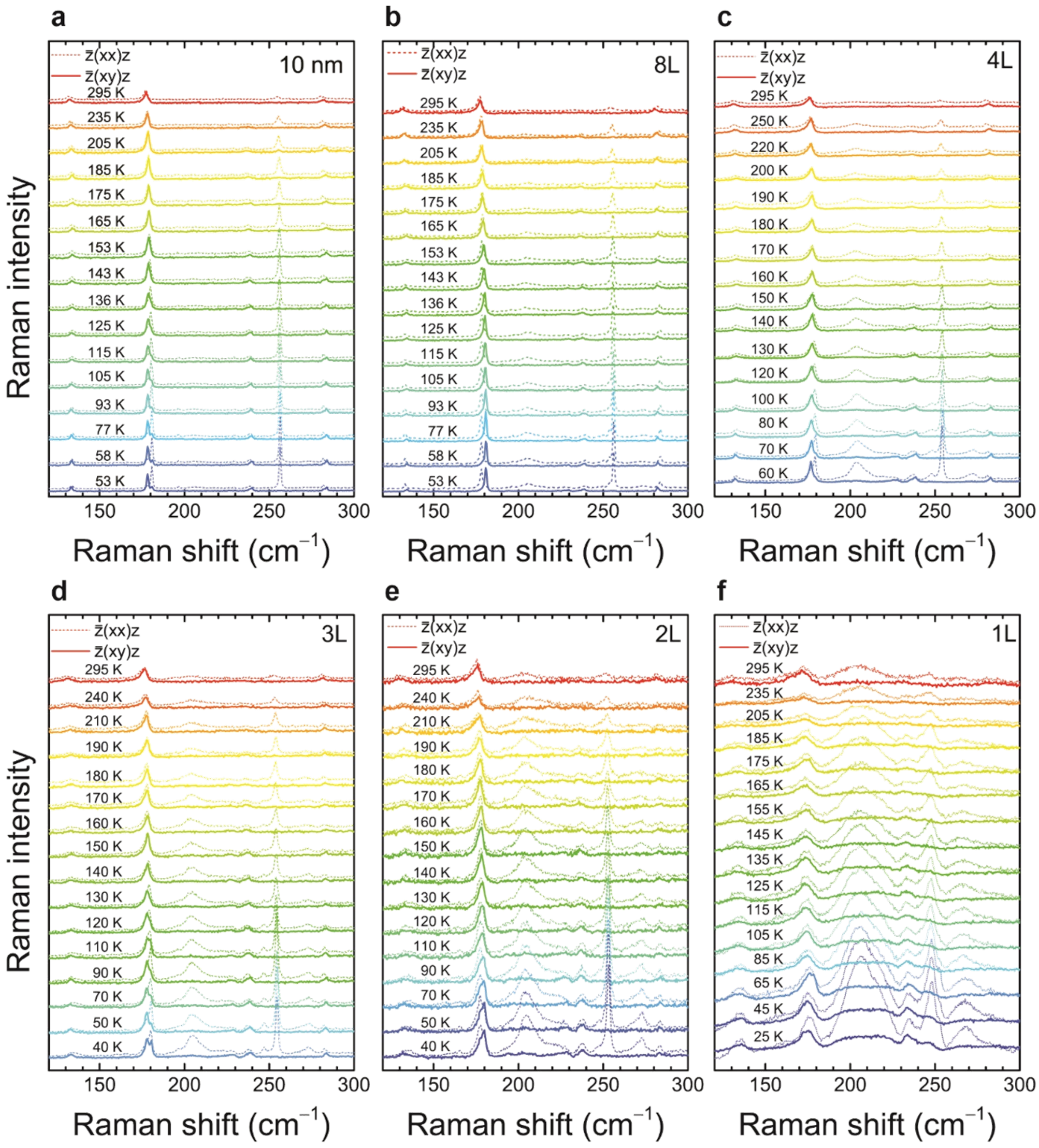

Supplementary Figure 11 | Temperature dependence of ΔP$_2$. a–f, Polarized Raman spectra of 10 nm (**a**), 8L (**b**), 4L (**c**), 3L (**d**), 2L (**e**), and 1L (**f**) $NiPS_3$ obtained by using parallel- and cross-polarization configurations as a function of temperature.

- **Supplementary Note 3.**

Extracting transition temperature by using ΔP_2

Baltensperger and Helman[16] developed a general theory of the spin-induced phonon frequency shift in magnetic crystals. The Hamiltonian in a magnetic crystal is expressed by $H = H^l + H^m$, where H^l is the pure lattice energy including anhanrmonic terms and H^m is the spin dependent phonon energy, expressed by

$$H_m = \sum_{i<j} I_{ij} S_i \cdot S_j \ , \tag{3}$$

where I_{ij} is the superexchange coupling constant between magnetic ions i, j; and S the ion spin operator. By solving the Hamiltonian, we can obtain the spin-induced phonon frequency which is simply expressed by $\Delta\omega \sim \langle S_0 \cdot S_1 \rangle / S^2$, where $\langle S_0 \cdot S_1 \rangle / S^2$ is the nearest neighbor spin correlation function. Near the phase transition temperature, the spin correlation function can be approximated as[17,18]

$$\langle S_0 \cdot S_1 \rangle / S^2 \sim m^2(T) \sim (T_\mathrm{N} - T)^{2\beta} \ . \tag{4}$$

Therefore, the difference of phonon energy between P_2 is simply expressed by

$$\Delta\omega_{P_2} \sim \left| M \langle S_0 \cdot S_1 \rangle / S^2 \right| \sim A(T_\mathrm{N} - T)^{2\beta} \ . \tag{5}$$

By assuming that the critical exponent is the same regardless of thickness, the magnetic transition temperature and the critical exponent can be estimated by fitting the data to Eq. (5). This yields $\beta \sim 0.16$ which is consistent with neutron scattering results[4] on bulk $NiPS_3$. In Supplementary Fig. 12, the experimental data and the fitting curves are compared.

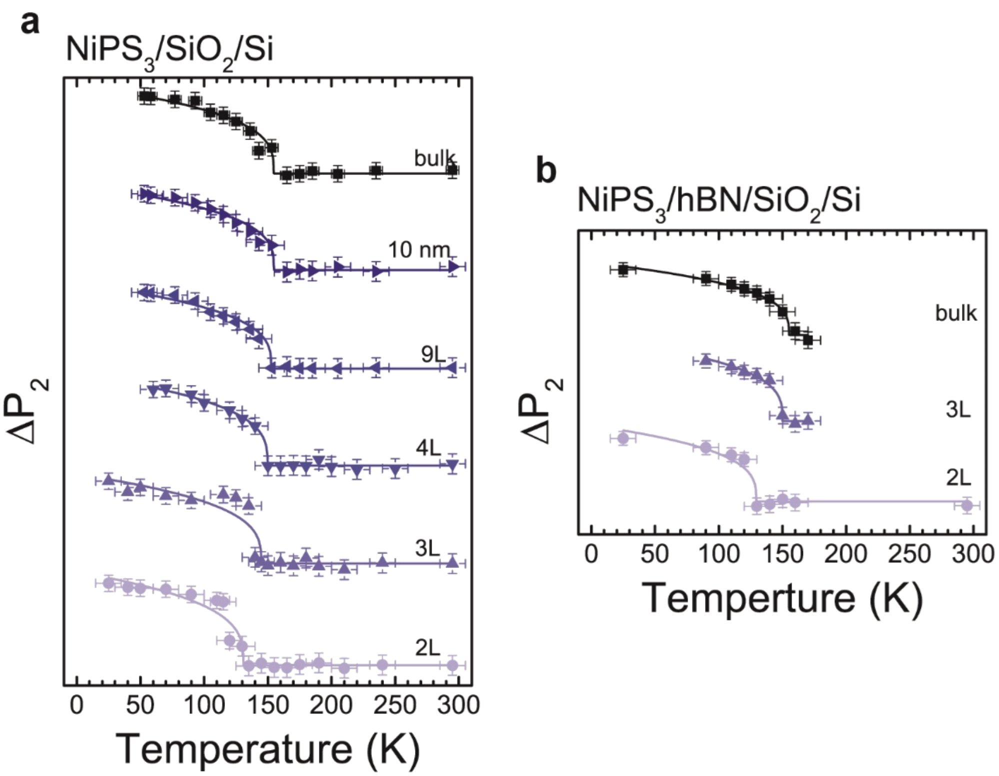

Supplementary Figure 12 | Extracting transition temperature by using ΔP_2. a,b, Temperature dependence of ΔP_2 for various thicknesses for $NiPS_3/SiO_2/Si$ samples (**a**), and $NiPS_3$/hBN/SiO_2/Si samples (**b**). The curves are fitting to Supplementary Eq. (5), and the error bars indicate experimental uncertainties.

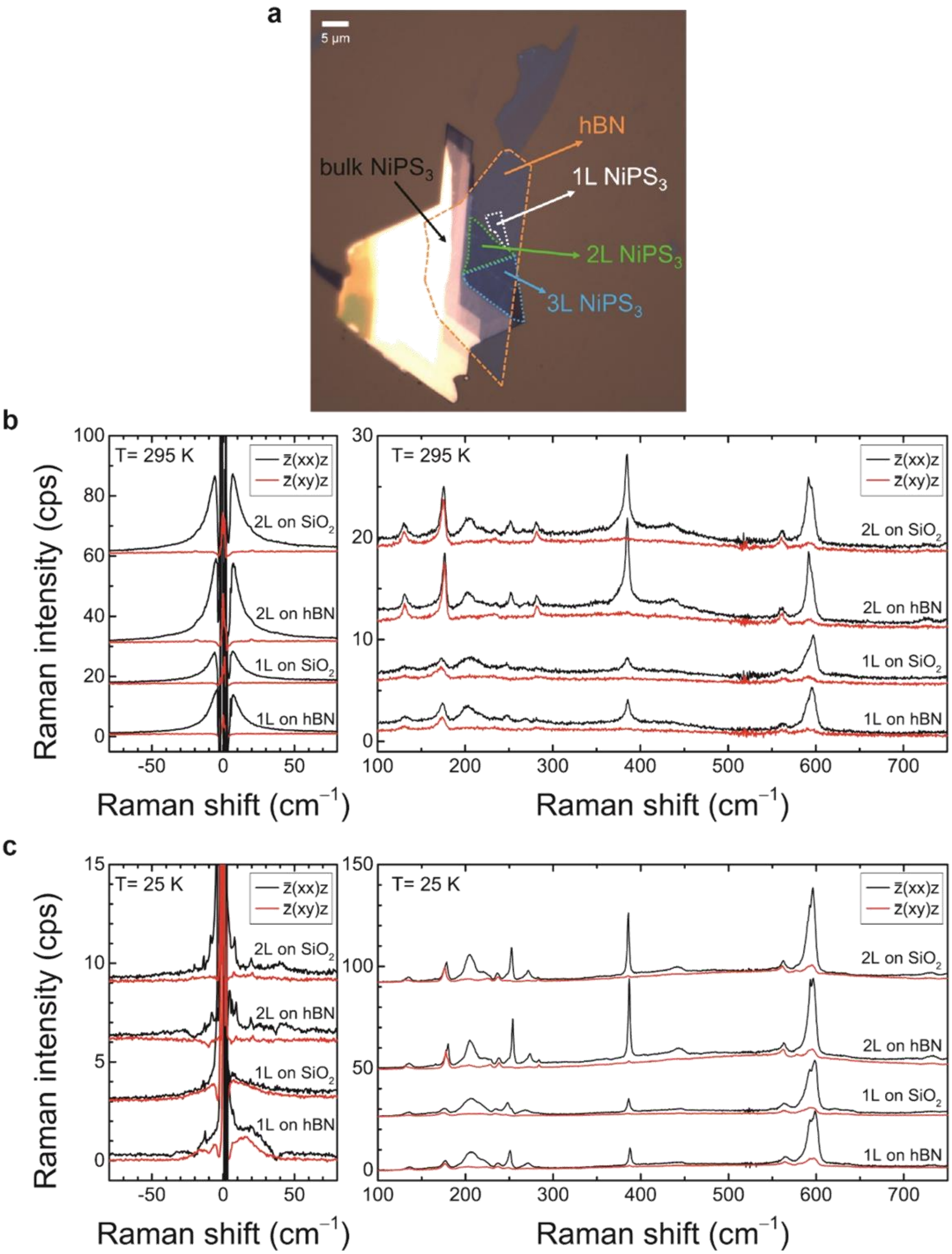

Supplementary Figure 13 | Comparison of substrate effects. a, Optical image of $NiPS_3$ exfoliated on hBN flake on SiO_2/Si. **b,c,** Polarized Raman spectra of $NiPS_3$ on SiO_2 and $NiPS_3$ on hBN at T=295 K (**b**) and T=25 K (**c**). No discernible differences are observed between two substrates.

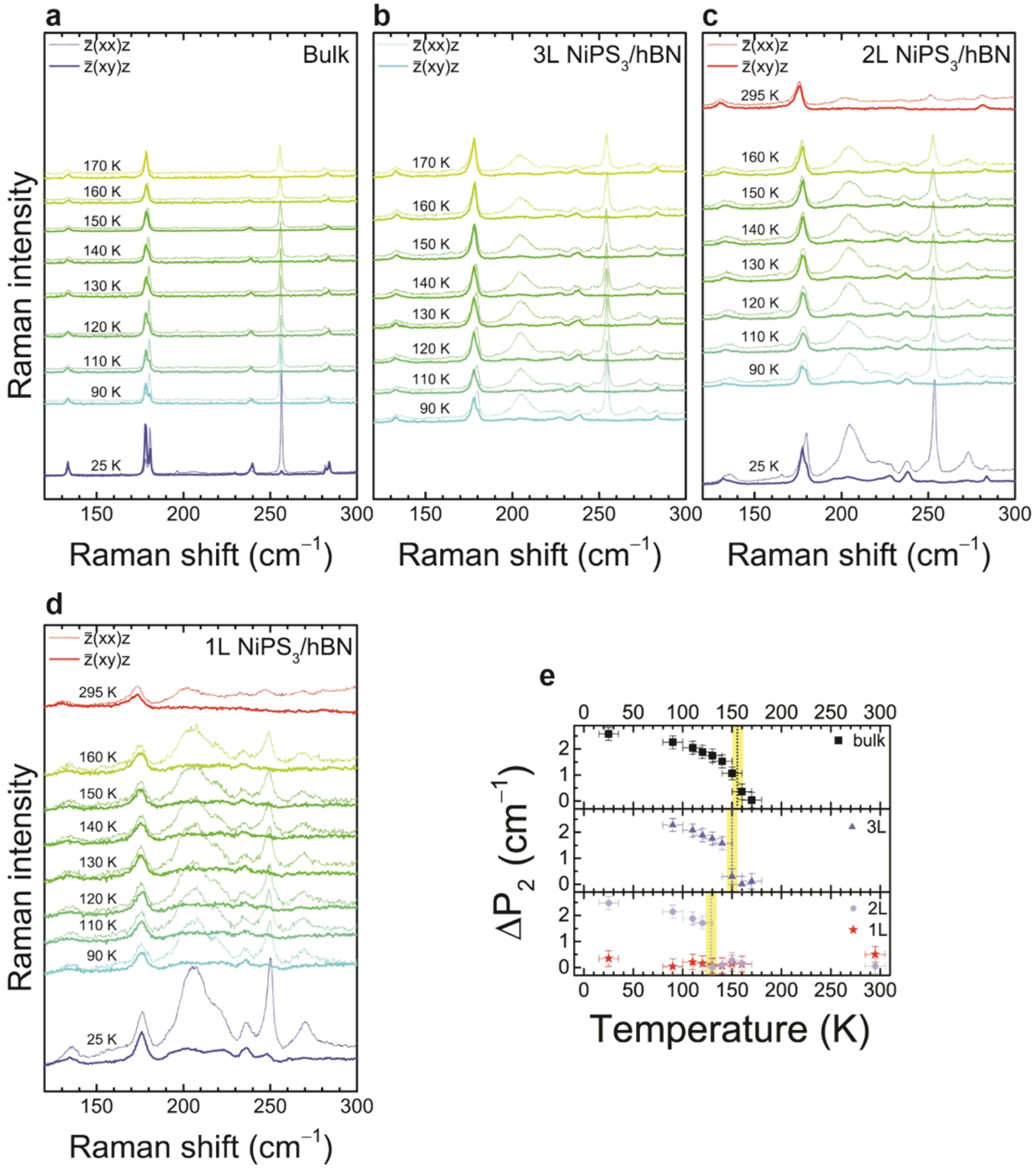

Supplementary Figure 14 | ΔP_2 in $NiPS_3$ on hexagonal boron nitride (hBN). a-d, Temperature dependent polarized Raman spectra of bulk (**a**), 3L (**b**), 2L (**c**), 1L (**d**) $NiPS_3$ on hBN. **e,** Temperature dependence of ΔP_2 for various thicknesses. The Error bars indicate experimental uncertainties. Dashed vertical lines show the Néel temperature for each thickness in **e**.

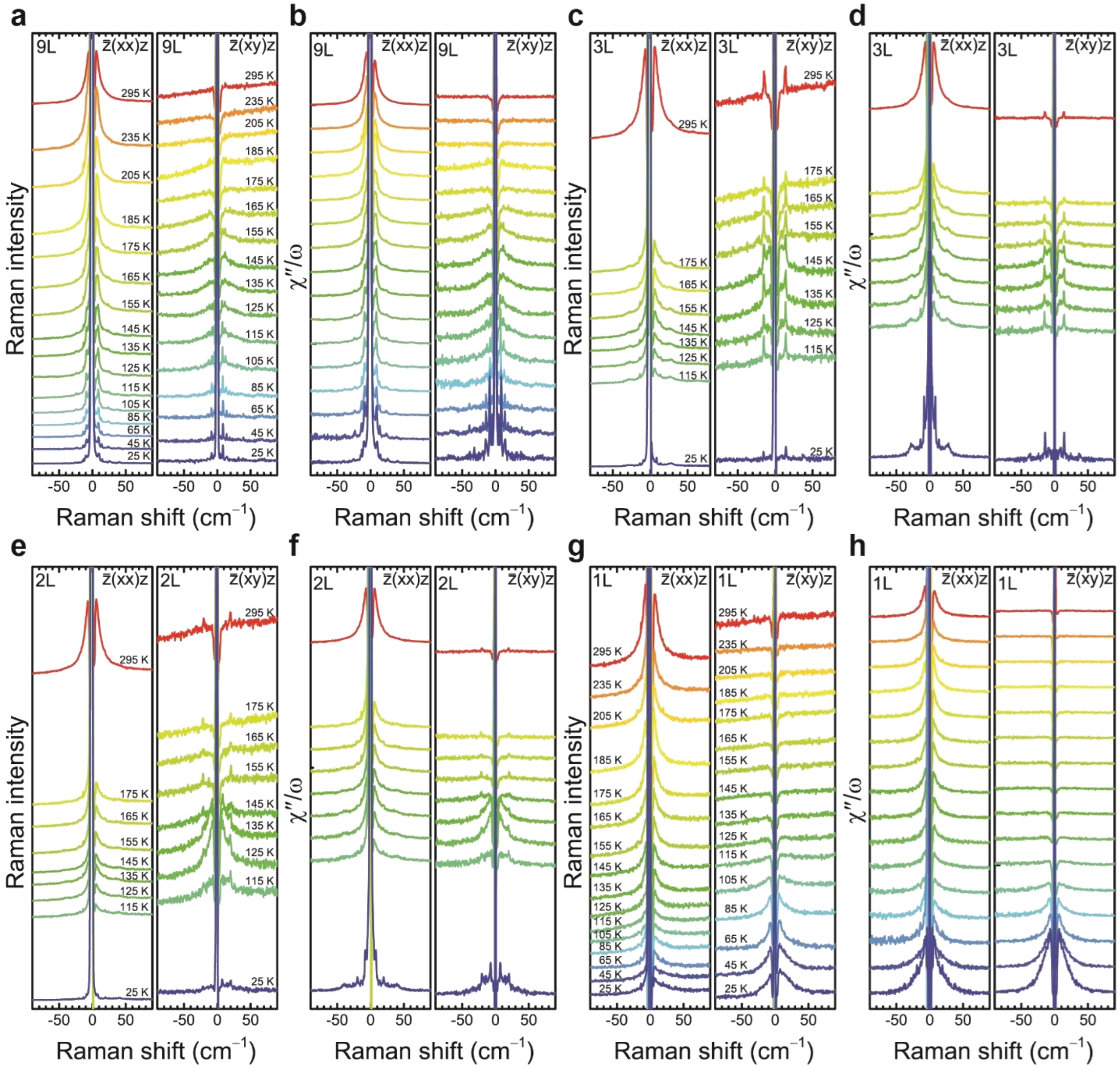

Supplementary Figure 15 | Temperature dependence of quasi-elastic scattering signals. Low-frequency Raman spectra (**a**, **c**, **e**, **g**) and Bose-Einstein-factor-corrected Raman response $\chi''(\omega)/\omega$ (**b**, **d**, **f**, **h**) for parallel- and cross-polarization configurations from 9L to 1L $NiPS_3$.

- **Supplementary Note 4.**

Monte Carlo simulations

We performed Monte Carlo simulations to calculate the physical quantities in N_z layers of stacked honeycomb lattice of spins. Each layer contains $N \times N$ honeycombs. The spin system is described by the Hamiltonian:

$$
\begin{aligned}
H = & J_1 \sum_{\langle i,j \rangle} \left[S_i^x S_j^x + S_i^y S_j^y + \alpha S_i^z S_j^z \right] + J_2 \sum_{\langle\langle i,k \rangle\rangle} \left[S_i^x S_k^x + S_i^y S_k^y + \alpha S_i^z S_k^z \right] \\
& + J_3 \sum_{\langle\langle\langle i,l \rangle\rangle\rangle} \left[S_i^x S_l^x + S_i^y S_l^y + \alpha S_i^z S_l^z \right] + J' \sum_{[i,m]} \left[S_i^x S_m^x + S_i^y S_m^y + \alpha S_i^z S_m^z \right],
\end{aligned} \tag{6}
$$

where single, double, and triple angular brackets in the sums denote nearest, next-nearest, third-nearest neighbors on the same plane, respectively, while square brackets denote nearest neighbors along the stacking direction.

We have performed Monte Carlo simulations to examine thermodynamic properties for three-dimensional stacked honeycomb lattice by using $N = N_z$. We have computed zigzag magnetization m_z defined by

$$
m_z = \frac{1}{2N^2 N_z} \left| \sum_i (-1)^{C_i} \mathbf{S}_i \right|, \tag{7}
$$

where C_i is the index of the chain to which the spin $\mathbf{S}_i$ belongs. We also calculate zero-field magnetic susceptibility χ,

$$
\chi \equiv \sum_{\alpha = x,y,z} \left. \frac{\partial m_\alpha}{\partial H_\alpha} \right|_{H \to 0^+}, \tag{8}
$$

with magnetic field $\mathbf{H}$ and magnetization

$$
\mathrm{m} \equiv \frac{1}{2N^2 N_z} \sum_i \langle \mathrm{S}_i \rangle. \tag{9}
$$

We have used parameters $J_1 S(S+1) = 10.3\, meV$, $J_2 = J_3 = J_1$, $J' = -J_1$, $\alpha = 0.66$; the inter-layer coupling is included for the bulk and the few-layer computations, and the intra-layer couplings are simplified due to computational costs, which do not affect qualitatively the Monte Carlo results. Under these parameters the spin system undergoes the phase transition into a magnetically ordered state with zigzag magnetic order at $T_{\mathrm{N}} \approx 155\,\mathrm{K}$.

With these exchange couplings we examined the properties of few layers of honeycomb lattice through Monte Carlo simulations. The simulations have been performed up to the size $N = 128$ for $N_z = 1, 2, 3, 4$. In Supplementary Fig. 16b, we plot zigzag magnetization at $T = 60\,\mathrm{K}$ as a function of N . The linear behavior in the log-log plot demonstrates well the power-law decrease with N and the resulting powers are close to two at high temperatures. As the temperature decreases the power decreases below a certain temperature, and approaches zero. By using the best linear fit, we have obtained zigzag magnetization m_z for $N = 10000$, which corresponds to typical sizes of samples in experiments. The plot of m_z versus N_z in Supplementary Fig. 16c demonstrates well that the zigzag magnetization for $N_z = 1$ is reduced in large systems much lower in comparison with $N_z > 1$. In Supplementary Fig. 16d we also plot the temperature dependence of extrapolated m_z for $N_z = 1, 2, 3$ together with that of three-dimensional systems. We observe that the onset temperature of m_z decreases monotonically with the decrease of N_z , which exhibits qualitative agreement with the experimental results in few layers of $NiPS_3$.

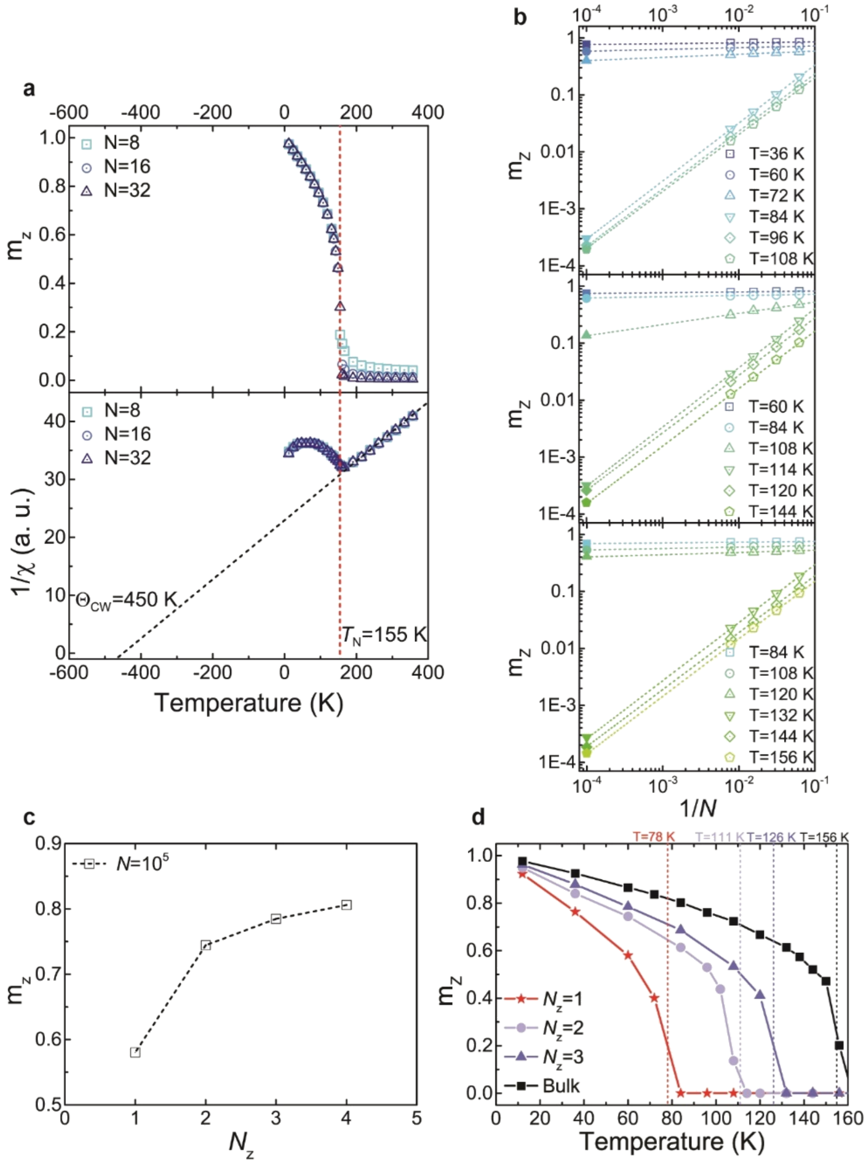

Supplementary Figure 16 | Monte Carlo simulation results. a, Zigzag magnetization m_z and inverse magnetic susceptibility $1/\chi$ as a function of temperature *T* in a three-dimensional stacked honeycomb lattice. The onset of zigzag magnetization (red line) occurs at $T_{\mathrm{N}} = 155\,\mathrm{K}$ and the extrapolation of high-temperature part of inverse magnetic susceptibility (green line) gives Curie temperature $\Theta_{\mathrm{CW}} = 450\,\mathrm{K}$. **b,** Log-log plot of zigzag magnetization m_z for $N_z = 1$ (top), 2 (middle), 3 (bottom) layers of honeycomb lattice as a function of lattice N at various temperatures *T*. Lines are best linear fits of the zigzag magnetization for each N_z and *T*. Filled symbols are extrapolated values at $N = 10000$. **c,** Zigzag magnetization m_z extrapolated to typical size $N = 10000$ as a function of the number of layers N_z at *T*=60 K. **d,** Zigzag magnetization extrapolated to $N = 10000$ as a function of temperature T for $N_z = 1, 2, 3$ layers and three-dimensional bulk lattice.

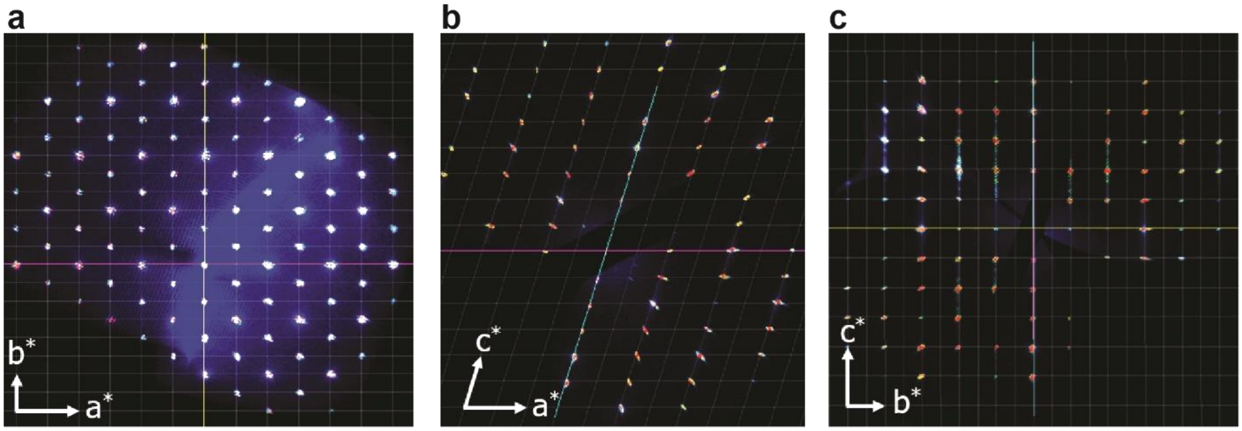

Supplementary Figure 17 | X-ray diffraction patterns of $NiPS_3$ single crystal. a-c, Bragg peaks of bulk $NiPS_3$ mapped on the reciprocal lattice for *ab* (**a**), *ac* (**b**), and *bc* (**c**) planes.

- **Supplementary Note 5.**

Degradation test for few-layer $NiPS_3$

Exfoliated few-layer $NiPS_3$ samples are relatively stable but show photo-degradation when the sample are exposed to focused laser in ambient conditions. To check that the few-layer samples are stable under the experimental conditions, we performed the degradation test of few-layer $NiPS_3$ samples as follow.

First, we checked photo-degradation of $NiPS_3$ in ambient air. We exposed a 2L $NiPS_3$ sample to focused laser beams with several different powers in ambient air for 1 min and obtained optical and atomic force microscopy (AFM) images (see Supplementary Fig. 18a). Some photo-degradation was observed on sample surfaces exposed to the laser. The degradations are more readily observed in the phase contrast image of AFM. Some degradation is observed from a spot exposed to as low-power as a 50-μW laser beam. Next, we performed similar tests for 1L and 2L $NiPS_3$ samples in vacuum (see Supplementary Figs. 18b and c). The samples were exposed to focused laser beams with several different powers for more than 30 min. There is no discernible change in the optical images. In AFM images, minor degradations can be observed from spots if the power of laser is higher than 150 μW. Since the power of the laser we used in our experiment was 100 μW, we can assume that photo-degradation should be minimal.

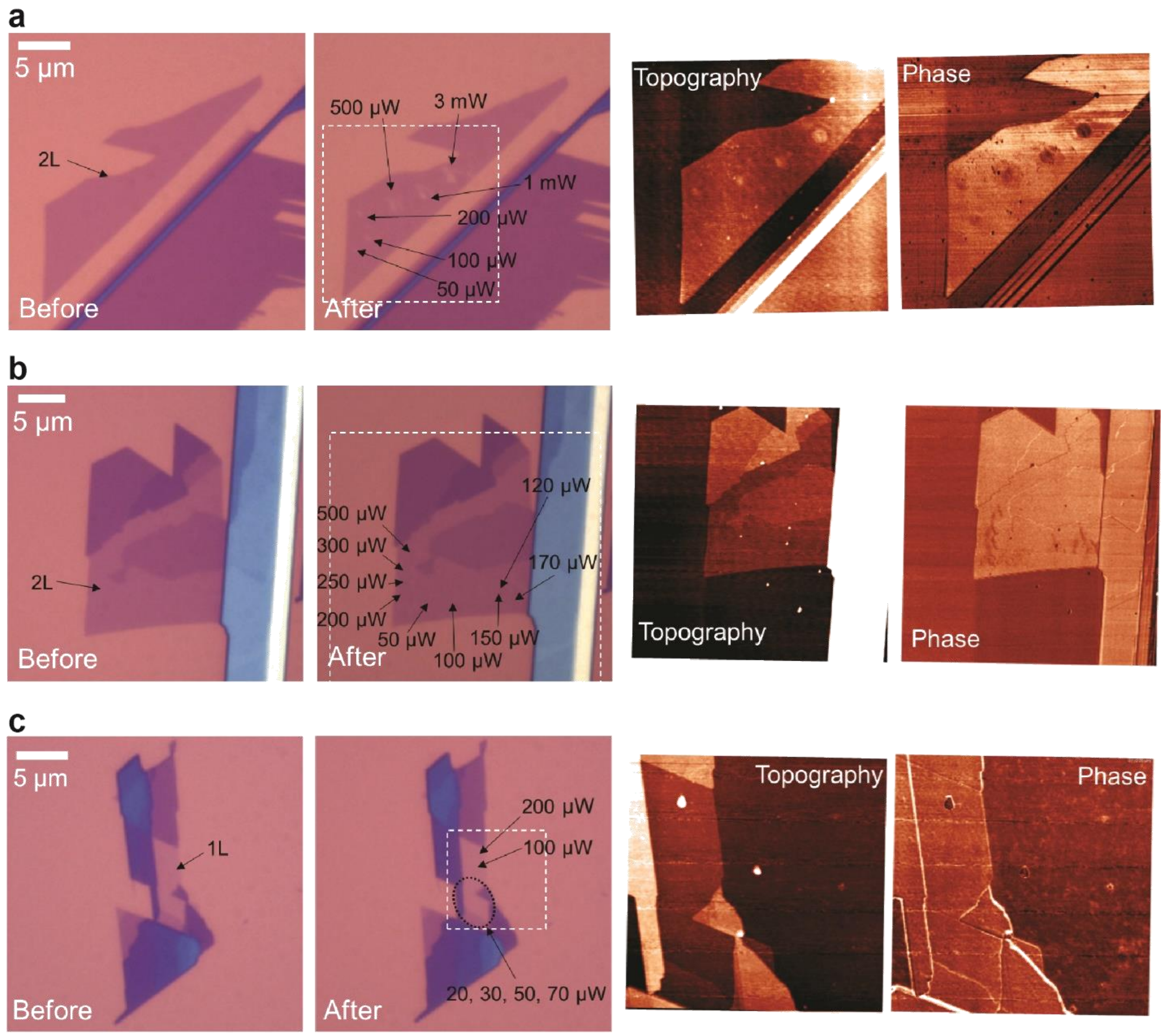

Supplementary Figure 18 | Photo-degradation test for few-layer $NiPS_3$. Optical images before and after exposing to several laser powers and atomic force microscopy images of topography and phase after exposing to laser of 2L $NiPS_3$ in ambient air (**a**), and 2L (**b**) and 1L $NiPS_3$ in vacuum (**c**).

Supplementary References.

1. Zhao, Y. *et al.* Interlayer Breathing and Shear Modes in Few-Trilayer MoS_2 and WSe_2. *Nano Lett.* **13,** 1007–1015 (2013).

2. Zhang, X. *et al.* Raman spectroscopy of shear and layer breathing modes in multilayer MoS_2. *Phys. Rev. B* **87,** 115413 (2013).

3. Brec, R. Review on structural and chemical properties of transition metal phosphorous trisulfides MPS_3. *Solid State Ionics* **22,** 3–30 (1986).

4. Wildes, A. R. *et al.* Magnetic structure of the quasi-two-dimensional antiferromagnet $NiPS_3$. *Phys. Rev. B* **92,** 224408 (2015).

5. Fouet, J. B., Sindzingre, P. & Lhuillier, C. An investigation of the quantum J_1-J_2-J_3 model on the honeycomb lattice. *Eur. Phys. J. B* **20,** 241–254 (2001).

6. Mulder, A., Ganesh, R., Capriotti, L. & Paramekanti, A. Spiral order by disorder and lattice nematic order in a frustrated Heisenberg antiferromagnet on the honeycomb lattice. *Phys. Rev. B* **81,** 214419 (2010).

7. Bishop, R. F., Li, P. H. Y., Farnell, D. J. J. & Campbell, C. E. The frustrated Heisenberg antiferromagnet on the honeycomb lattice: J_1–J_2 model. *J. Phys. Condens. Matter* **24,** 236002 (2012).

8. Li, P. H. Y., Bishop, R. F. & Campbell, C. E. Phase diagram of a frustrated spin-1/2 J_1-J_2 XXZ model on the honeycomb lattice. *Phys. Rev. B* **89,** 220408 (2014).

9. Ressouche, E. *et al.* Magnetoelectric $MnPS_3$ as a candidate for ferrotoroidicity. *Phys. Rev. B* **82,** 100408 (2010).

10. Lançon, D. *et al.* Magnetic structure and magnon dynamics of the quasi-two-dimensional antiferromagnet $FePS_3$. *Phys. Rev. B* **94,** 214407 (2016).

11. Nguyen, T. M. H. *et al.* Two-magnon scattering in the 5d all-in-all-out pyrochlore magnet $Cd_2Os_2O_7$. *Nat. Commun.* **8,** 251 (2017).

12. Toth, S. & Lake, B. Linear spin wave theory for single-Q incommensurate magnetic structures. *J. Phys. Condens. Matter* **27,** 166002 (2015).

13. del Corro, E. *et al.* Atypical Exciton–Phonon Interactions in WS_2 and WSe_2 Monolayers Revealed by Resonance Raman Spectroscopy. *Nano Lett.* **16,** 2363–2368 (2016).

14. Carvalho, B. R. *et al.* Intervalley scattering by acoustic phonons in two-dimensional MoS_2 revealed by double-resonance Raman spectroscopy. *Nat. Commun.* **8,** 14670 (2017).

15. Bernasconi, M. *et al.* Lattice dynamics of layered MPX_3 (M=Mn,Fe,Ni,Zn; X=S,Se) compounds. *Phys. Rev. B* **38,** 12089–12099 (1988).

16. Baltensperger, W. & Helman, J. S. Influence of magnetic order in insulators on the optical phonon frequency. *Helv. Phys. Acta* **41,** 668–673 (1968).

17. Callen, E. Optical Absorption Edge of Magnetic Semiconductors. *Phys. Rev. Lett.* **20,** 1045–1048 (1968).

18. Ashcroft, N. W. & Mermin, N. D. *Solid State Physics*. (Brooks/Cole, 1976).
